# Pericapsular Nerve Group Block for Prosthetic Hip Reduction in the Emergency Department: Case Report

**DOI:** 10.5811/cpcem.21137

**Published:** 2024-12-17

**Authors:** Rebecca Thomas, Matthew Carr, Janae Fry, Taryn Hoffman

**Affiliations:** HCA Orange Park Hospital, Emergency Department, Orange Park, Florida

**Keywords:** PENG block, femoral nerve block, fascia iliaca block, case report

## Abstract

**Introduction:**

A pericapsular nerve group (PENG) block is unique compared to other regional anesthetic techniques (femoral nerve and fascia iliaca blocks) because it is a motor-sparing block. It also provides anesthesia to more nerves that innervate the anterior capsule of the femoroacetabular joint when compared to the femoral nerve and fascia iliaca blocks. Additionally, regional anesthesia carries fewer risks and requires less resources when compared with procedural sedation, which is the typical method for reducing a dislocated femoroacetabular joint.

**Case Report:**

We describe a novel case in which a PENG block was used in the emergency department (ED) to reduce a prosthetic hip dislocation.

**Conclusion:**

The PENG block is a safe and effective method of achieving sufficient analgesia to reduce prosthetic hips in the ED.

## INTRODUCTION

The incidence of hip dislocation after undergoing total hip arthroplasty (THA) ranges from 1–10%.^1^,^2^ The conventional treatment for prosthetic hip dislocation is either reduction under procedural sedation in the emergency department (ED) or general anesthesia in the operating room.^3^,^4^ However, procedural sedation is resource-intensive and is associated with multiple complications, including respiratory depression, hypotension, adrenal insufficiency, and immunosuppression.^5^–^7^

The pericapsular nerve group (PENG) block was first described in the literature in 2018 by Giron et al, as a means to provide pre- and postoperative analgesia to patients with proximal femur fractures.^8^ As a relatively new procedure, literature demonstrating its use in the ED is limited and to our knowledge has yet to be reported in the management of acute prosthetic hip dislocation. We describe a case of a patient with recurrent prosthetic hip dislocations who underwent successful reduction in the ED using only the PENG block on three separate visits.

## CASE REPORT

An 81-year-old male with a past medical history significant for hypertension, congestive heart failure, and surgical history of three-vessel coronary artery bypass grafting and left THA in 1996 presented to the ED for evaluation of left hip pain after bending over to tie his shoe. The patient had a history of numerous hip dislocations. Revision THA was attempted in 2021; however, the patient suffered cardiac arrest upon general anesthesia induction, and the procedure was aborted. Hip radiographs obtained in the ED were remarkable for a posterior dislocation of the left prosthetic hip. The patient received acetaminophen by mouth, and a PENG block was performed with sufficient analgesia for the left hip to be reduced by the Allis maneuver without any complications. He was discharged home with a knee immobilizer and standard posterior hip dislocation precautions. The door-to-disposition time for this encounter was 4.5 hours. Subsequently, this patient presented with a left hip dislocation on two additional occasions, and the left hip was successfully reduced during these two other encounters using the PENG block and either acetaminophen or ketorolac. He provided written authorization for his information to be released in this case report.

## DISCUSSION

The use of regional anesthesia first emerged in the emergency medicine literature in the 1980s with articles demonstrating the use of femoral nerve blocks to provide analgesia for proximal femur fractures.^9^ Multiple studies have since illustrated the benefits of regional analgesia compared to systemic analgesia, including reduced mortality rate, decreased length of hospital stay, and lower pain scores.^10^,^11^ Historically, the most common way to achieve analgesia of the proximal femur was with a femoral nerve or fascia iliaca compartment block.^12^

A fascia iliaca block involves injecting local anesthetic into the fascia iliaca compartment, which contains both the femoral and lateral femoral cutaneous nerves.^13^ However, the anterior femoroacetabular joint capsule receives sensory innervation from the femoral, obturator, and accessory obturator nerves.^14^ So, the fascia iliaca and femoral nerve blocks do not provide complete analgesia when attempting to anesthetize the femoroacetabular joint. Furthermore, these two nerve blocks affect the motor fibers of the femoral nerve, resulting in weakness of knee extension.

The PENG block is a relatively new, regional anesthetic technique developed to provide more complete analgesia of the terminal nerves that innervate the femoroacetabular joint capsule.^13^ This is an interfascial plane block where local anesthetic is deposited between the psoas tendon and the ileum to achieve analgesia of the terminal branches of the three nerves that provide sensation to the anterior femoroacetabular joint capsule, demonstrated in the Image.^13^ An additional benefit of the PENG block is that it is motor-sparing and, therefore, ideal for patients who will be discharged from the ED. However, the motor-sparing effects of the PENG block can also be considered a disadvantage as there is more muscle relaxation achieved with the fascia iliaca block. For patients who are experiencing their first prosthetic hip dislocation, more muscle relaxation may be required for hip reduction than for patients who have a history of recurrent dislocations.

**CPC-EM Capsule**

What do we already know about this clinical entity?

*Pericapsular nerve group (PENG) blocks provide local analgesia to the proximal femur and have historically been used for pain control for proximal femur fractures*.

What makes this presentation of disease reportable?

*We describe a novel case in which a PENG block was used in the emergency department (ED) for local analgesia for prosthetic hip reduction*.

What is the major learning point?

*A PENG block is a safe and effective method of achieving sufficient analgesia to reduce prosthetic hips in the ED*.

How might this improve emergency medicine practice?

*Using a PENG block for hip reduction is less resource intensive than moderate sedation and is motor-sparing, which is ideal for patients who will be discharged from the ED*.

The technique for performing a PENG block is as follows:^13^,^15^

Using a low-frequency curvilinear probe with the probe indicator facing the patient’s right side, place the probe parallel to and just inferior to the patient’s inguinal ligament. First, identify the head of the femur, and then slide the probe cranially and medially until you obtain a view of both the anterior inferior iliac spine and the iliopubic eminence as seen in the image.

After anatomic landmarks have been identified, clean and drape the region using standard sterile technique, including a sterile probe cover. An 8–10 centimeter 20–22 gauge needle should then be advanced from lateral-to-medial using in-plane needle visualization to reach the target area between the psoas tendon and ilium. After negative aspiration, 15–20 milliliters of local anesthetic (ideally an agent with a longer half-life such as bupivacaine) is deposited below the psoas tendon.

Contraindications to performing a PENG block include patient allergy to local anesthetic, inability to visualize the anatomic landmarks, and infection overlying the injection site. Complications of the PENG block include infection at the injection site, failure of the block to provide sufficient analgesia, damage to local structures, and intravascular injection of local anesthetic resulting in systemic toxicity.

## CONCLUSION

This case demonstrates an expansion to the indications for a pericapsular nerve group block in the ED. The PENG block is a safe and effective method of achieving sufficient analgesia to reduce prosthetic hips in the ED. This method of hip reduction eliminates the adverse effects of procedural sedation and the necessity for resource-intensive sedation in the ED.

## Figures and Tables

**Image:**
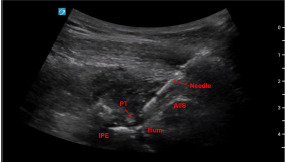
Pericapsular nerve group nerve block landmarks. Needle advances in a lateral-to-medial direction toward the ilium near the psoas tendon (PT), between the anterior inferior iliac spine (AIIS) laterally, and the iliopubic eminence (IPE) medially.
